# Update on the human and mouse lipocalin (*LCN*) gene family, including evidence the mouse *Mup* cluster is result of an “evolutionary bloom”

**DOI:** 10.1186/s40246-019-0191-9

**Published:** 2019-02-19

**Authors:** Georgia Charkoftaki, Yewei Wang, Monica McAndrews, Elspeth A. Bruford, David C. Thompson, Vasilis Vasiliou, Daniel W. Nebert

**Affiliations:** 1 Department of Environmental Health Sciences, Yale School of Public Health, Yale University, New Haven, CT 06520-8034 USA; 2 Mouse Genome Informatics, The Jackson Laboratory, 600 Main Street, Bar Harbor, ME 04609 USA; 3 HUGO Gene Nomenclature Committee, European Bioinformatics Institute, Wellcome Genome Campus, Hinxton, Cambridge, CB10 1SD UK; 4 Department of Clinical Pharmacy, Skaggs School of Pharmacy and Pharmaceutical Sciences, University of Colorado, Aurora, CO USA; 5 Department of Environmental Health and Center for Environmental Genetics; Department of Pediatrics and Molecular and Developmental Biology, Cincinnati Children’s Research Center, University Cincinnati Medical Center, Cincinnati, OH 45267 USA

## Abstract

Lipocalins (LCNs) are members of a family of evolutionarily conserved genes present in all kingdoms of life. There are 19 *LCN*-like genes in the human genome, and 45 *Lcn*-like genes in the mouse genome, which include 22 major urinary protein (*Mup*) genes. The *Mup* genes, plus 29 of 30 *Mup-ps* pseudogenes, are all located together on chromosome (Chr) 4; evidence points to an “evolutionary bloom” that resulted in this *Mup* cluster in mouse, syntenic to the human Chr 9q32 locus at which a single *MUPP* pseudogene is located. LCNs play important roles in physiological processes by binding and transporting small hydrophobic molecules —such as steroid hormones, odorants, retinoids, and lipids—in plasma and other body fluids. LCNs are extensively used in clinical practice as biochemical markers. LCN-like proteins (18–40 kDa) have the characteristic eight β-strands creating a barrel structure that houses the binding-site; LCNs are synthesized in the liver as well as various secretory tissues. In rodents, MUPs are involved in communication of information in urine-derived scent marks, serving as signatures of individual identity, or as kairomones (to elicit fear behavior). MUPs also participate in regulation of glucose and lipid metabolism via a mechanism not well understood. Although much has been learned about LCNs and MUPs in recent years, more research is necessary to allow better understanding of their physiological functions, as well as their involvement in clinical disorders.

## Introduction

Lipocalins (LCNs) are members of a family that includes a diverse group of low-molecular-weight (18–40 kDa) proteins. The larger members of this family undergo cleavage to form the ultimate LCN protein. Comprising usually 150–180 amino-acid residues, these proteins belong to the *calycin superfamily* and are widely dispersed throughout all kingdoms of life [1]. LCNs are evolutionarily conserved and share an eight-stranded antiparallel β-sheet structure; this forms a “barrel” which is the internal ligand-binding site that interacts with and transports small hydrophobic molecules—such as steroid hormones, odorants (e.g., pheromones), retinoids, and lipids [2, 3].

There are three main structurally conserved regions (SCR1, SCR2, SCR3) that are shared in the lipocalin fold; these represent a moiety composed of three loops that are close to each other in the three-dimensional structure of the β-strands that make up the barrel [4–7]. Based on the SRCs, two separate groups have been proposed: the kernel LCNs and the outlier LCNs [3]. The kernel LCNs represent a core set of proteins sharing the three characteristic motifs, while the outlier LCNs, which are more divergent family members, typically share only one or two motifs [4]. Based on this categorization—retinoic acid-binding protein-4 (RBP4), α_1_-microglobulin (A1M), apolipoprotein D (APOD), complement C8 gamma chain (C8G), prostaglandin D_2_ synthase (PTGDS), and the major urinary proteins (MUPs)—have all been classified as kernel lipocalins, while odorant-binding proteins (OBP2A, OBP2B) and von Ebner’s gland protein (LCN1) are included in the outlier category [4, 7].

Depending on the structure of the individual LCN, the binding-site pocket can accommodate molecules of various sizes and shapes—thus contributing to the diversity of functions within this protein family [5]. Lipocalin crystal structures confirm the highly conserved eight continuously-hydrogen-bonded antiparallel β-strand domains creating the barrel.

The fatty acid-binding protein (*FABP*) gene family is considered a related, but distinct, subfamily of the calycin superfamily [8] and will not be discussed further here. Another subset of the lipocalins worthy of mention is the immunocalin subfamily. These include α_1_-acid glycoprotein, α_1_-microglobulin/bikunin precursor, and glycodelin, each of which exert significant immunomodulatory effects in cell culture [9, 10]; interestingly, all three are encoded by genes in the human Chr 9q32-34 region—together with at least four other lipocalins (neutrophil gelatinase-associated lipocalin, complement factor γ-subunit, tear prealbumin, and prostaglandin D synthase), which also might exert anti-inflammatory and/or antimicrobial activity [11].

## Lipocalin family in humans

**Fig. 1:**
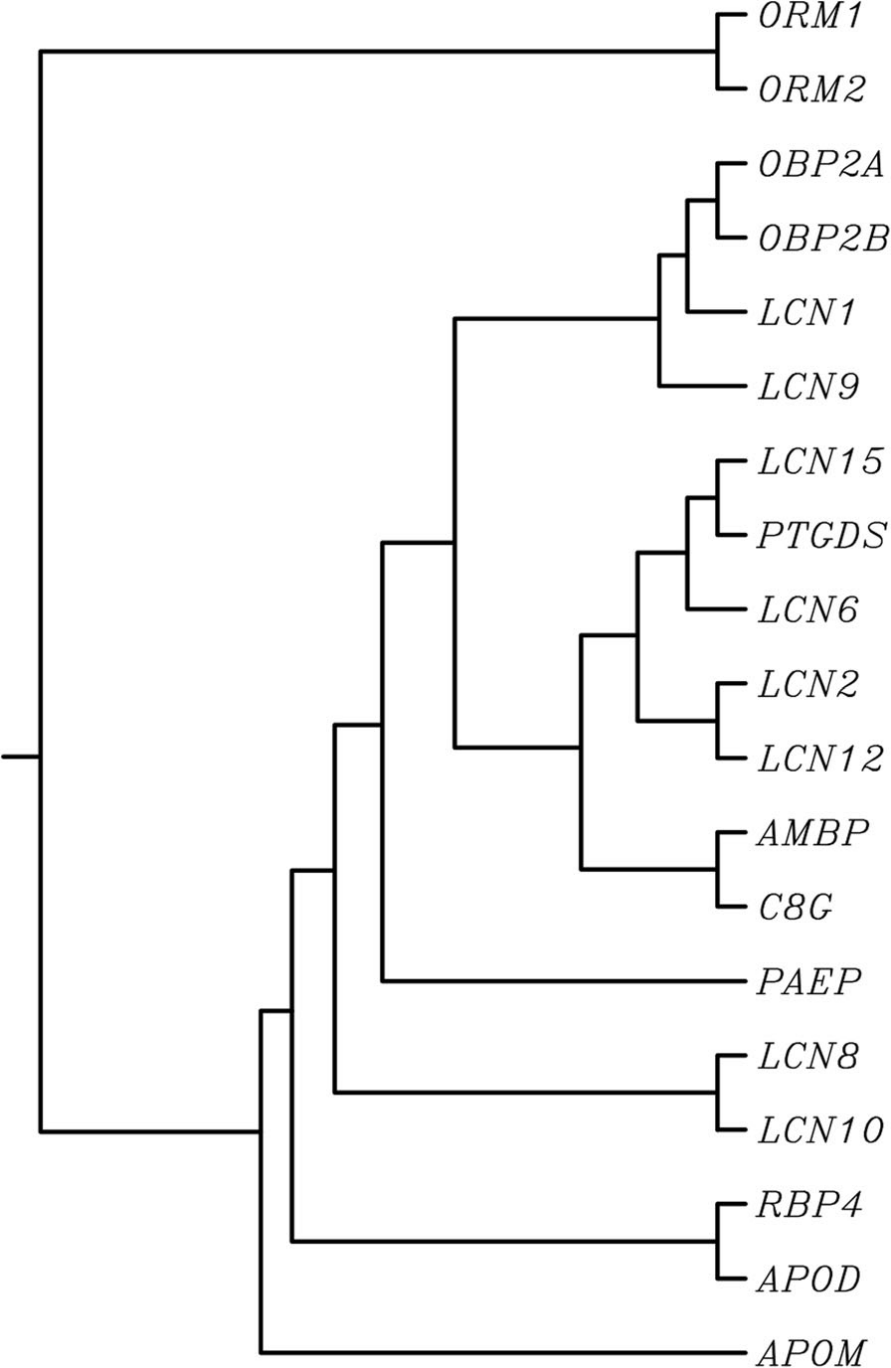
Dendrogram of lipocalins (LCNs) in the human genome. Although the names listed are the official human gene symbols [https://www.genenames.org/], this dendrogram is based on the alignment of proteins (listed in Table 1), using multiple sequence alignment by CLUSTALW (http://www.genome.jp/tools/clustalw/)

Among bacteria, plants, fungi, and animals, more than 1000 *LCN* genes have been identified to date. Nineteen *LCN* genes, encoding functional LCN proteins, exist in the human genome (Table 1). Figure 1 dendrogram shows the evolutionary relatedness of these human LCN proteins.

**Table 1:** List of all human *LCN* and mouse *Lcn* genes—with official gene symbols, full protein name, aliases, chromosomal locations, isoforms, National Center for Biotechnology Information (NCBI) RefSeq mRNA accession numbers, NCBI RefSeq protein accession numbers, and total number of amino acids (# of AAs) [information retrieved and confirmed from https://www.genenames.org/needs a close bracket]

Gene symbol	Full protein name	Aliases	Chromosome	Isoforms	Ref seq mRNA number	Ref seq protein number	No. of AAs
*LCN1*	Lipocalin-1 isoform 1 precursor	TP; TLC; PMFA; VEGP	9q34		NM_001252617.1	NP_001239546	176
	Lipocalin-1 isoform 2 precursor		9q34		NM_001252618.1	NP_001239547.1	233
	Lipocalin-1 isoform 3 precursor		9q34		NM_001252619.1	NP_001239548.1	230
*LCN2*	Lipocalin-2	24p3; MSFI; NGAL	9q34		NM_005564.3	NP_005555.2	198
*Lcn2*	Lipocalin-2	NRL; 24p3; Sip24; AW212229	2		NM_008491.1	NP_032517.1	200
*Lcn3*	Lipocalin-3	Vnsp1	2		NM_010694.1	NP_034824.1187	187
*Lcn4*	Lipocalin-4	Vnsp2; A630045M08Rik	2		NM_010695.1	NP_034825.1	185
*Lcn5*	Lipocalin-5	Erabp; MEP10; ERABP	2	Epididymal-specific lipocalin-5 isoform X1	XM_006497665.1	XP_006497728.1	208
				Epididymal-specific lipocalin-5 isoform X2	XM_006497666.3	XP_006497729.1	202
				Epididymal-specific lipocalin-5 isoform X3	XM_006497667.2	XP_006497730.1	190
				Epididymal-specific lipocalin-5 isoform X4	XM_006497668.2	XP_006497731.1	190
				Epididymal-specific lipocalin-5 isoform X5	XM_006497669.1	XP_006497732.1	173
*LCN6*	Lipocalin-6	LCN5; hLcn5; UNQ643	9q34.3		NM_198946.2	NP_945184.1	163
*Lcn6*	Lipocalin-6	9230101D24Rik	2	Epididymal-specific lipocalin-6 isoform 1 precursor;	NM_001276448.1	NP_001263377.1	245
				Epididymal-specific lipocalin-6 isoform 2 precursor	NM_177840.4	NP_808508.2	181
*LCN8*	Lipocalin-8	EP17; LCN5	9q34.3		NM_178469.3	NP_848564.2	152
*Lcn8*	Lipocalin-8	EP17; Lcn5; mEP17; 9230106L18Rik	2		NM_033145.1	NP_149157.1	175
*LCN9*	Lipocalin-9	HEL129; 9230102I19Rik	9q34.3		NM_001001676.1	NP_001001676.1	176
*Lcn9*	Lipocalin-9	9230102I19Rik	2		NM_029959.2	NP_084235.1	178
*LCN10*	Lipocalin-10		9q34.3		NM_001001712.2	NP_001001712.2	200
*Lcn10*	Lipocalin-10	9230112J07Rik	2		NM_178036.4	NP_828875.1	182
*Lcn11*	Lipocalin-11	Gm109	2		NM_001100455.2	NP_001093925.1	178
*LCN12*	Lipocalin-12		9q34.3		NM_178536.3	NP_848631.2	192
*Lcn12*	Lipocalin-12	9230102M18Rik	2		NM_029958.1	NP_084234.1	193
*LCN15*	Lipocalin-15	PRO6093; UNQ2541	9q34.3		NM_203347.1	NP_976222.1	184
*Lcn15*	Lipocalin-15	Gm33749	2 A3; 2		XM_006498514.1	XP_006498577.1	202
*Lcn16*	Lipocalin-16	Gm39773	2		XM_011239226.1	XP_011237528.1	181
*Lcn17*	Lipocalin-17	Gm39774	2		XM_011239227.1	XP_011237529.1	193
*OBP2A*	Odorant binding protein 2A	OBP; LCN13; OBP2C; OBPIIa; hOBPIIa	9q34		NM_001293189.1	NP_001280118.1	228
*Obp2a*	Odorant binding protein 2A	Lcn13; BC027556	2		NM_153558.1	NP_705786.1	176
*OBP2B*	Odorant-binding protein-2B	LCN14; OBPIIb	9q34		NM_001288987.1	NP_001275916.1	170
*Obp2b*	Odorant-binding protein-2B	Lcn14	2		NM_001099301.1	NP_001092771.1	176
*AMBP*	Alpha-1-microglobulin/bikunin precursor	A1M; HCP; ITI; UTI; EDC1; HI30; ITIL; IATIL; ITILC	9q32-q33		NM_001633.3	NP_001624.1	352
*Ambp*	Alpha 1 microglobulin/bikunin precursor	AI194774, ASPI, HI-30, Intin4, Itil, UTI	4 B3; 4 33.96 cM		NM_007443.4	NP_031469.1	349
*APOD*	Apolipoprotein D		3q29		NM_001647.3	NP_001638.1	189
*Apod*	Apolipoprotein D		16 B2; 16 21.41 cM		NM_001301353.1	NP_001288282.1	189
*APOM*	Apolipoprotein M	G3a; NG20; apo-M; HSPC336	6p21		NM_019101.2	NP_061974.2	188
*Apom*	Apolipoprotein M	G3a; NG20; 1190010O19Rik	17; 17 B1		NM_018816.1	NP_061286.1	190
*C8G*	Complement component 8, gamma polypeptide	C8C	9q34.3		NM_000606.2	NP_000597.2	202
*C8g*	Complement component 8, gamma polypeptide		2 A3; 2 17.31 cM		NM_001271777.1	NP_001258706.1	168
*ORM1*	Orosomucoid-1	ORM; AGP1; AGP-A; HEL-S-153w	9q32		NM_000607.2	NP_000598.2	201
*Orm1*	Orosomucoid-1	Agp-1; Agp-2; Orm-1	4 B3; 4 33.96 cM		NM_008768.2	NP_032794.1	207
*ORM2*	Orosomucoid-2	AGP2; AGP-B; AGP-B	9q32		NM_000608.2	NP_000599.1	201
*Orm2*	Orosomucoid-2	Agp1; Orm-2	4 B3; 4 33.96 cM		NM_011016.2	NP_035146.1	207
*PAEP*	Progestagen-associated endometrial protein	GD; GdA; GdF; GdS; PEP; PAEG; PP14	9q34		NM_001018049.1	NP_001018059.1	180
*PTGDS*	Prostaglandin D2 synthase; 21 kDa (brain)	PDS; PGD2; PGDS; LPGDS; PGDS2; L-PGDS	9q34.2-q34.3		NM_000954.5	NP_000945	190
*Ptgds*	Prostaglandin D2 synthase; 21 kDa (brain)	PGD2; PGDS; 21 kDa; PGDS2; Ptgs3; L-PGDS	2 A3; 2 17.28 cM		NM_008963.3	NP_032989.2	189
*RBP4*	Retinol-binding protein-4, plasma	RDCCAS; MCOPCB10	10q23.33		NM_006744.3	NP_006735.2	201
*Rbp4*	Retinol-binding protein-4, plasma	Rbp-4	19 C2; 19 32.75 cM		NM_001159487.1	NP_001152959.1	245

In humans, LCNs are located in blood plasma and other body fluids such as tears and genital secretions, in which they serve as carriers for a variety of small molecules [5]. LCNs also can play important roles in disease such as diabetic retinopathy [12], and, as a result, they are extensively used clinically as biochemical markers. For example, A1M (α_1_-microglobulin/bikunin precursor, encoded by the *AMBP* gene) is a biomarker of proteinuria and indicator of declining renal function [13].

Lipocalin-1 (LCN1; human tear pre-albumin, or von Ebner’s gland protein) is one of four major proteins in human tears, acting as a lipid sponge on the ocular surface [14, 15]. LCN1 is produced by lacrimal glands and secreted into tear fluid. Decreased LCN1 levels are associated with Sjögren’s disease, LASIK-induced dry-eye disease [16], and diabetic retinopathy [12].

Lipocalin-2 (LCN2; also known as neutrophil gelatinase-associated lipocalin) mediates various inflammatory processes by suppressing macrophage interleukin-10 (IL10) production [17, 18]. Several studies have shown that *LCN2* gene expression in adipose tissue is elevated in insulin-resistant states [19, 20]. LCN2 is also involved in kidney development and used as a biomarker for acute and chronic renal injury [21].

Odorant-binding proteins 2A and 2B (encoded by the *OBP2A* and *OBP2B* genes) are members of the LCN family. OBP2A is highly expressed in the oral sphere (e.g., nasal mucus, salivary, and lacrimal glands), whereas OBP2B is expressed in endocrine organs (e.g., mammary gland and prostate) [22]. Functioning as soluble-carrier proteins, OBP2A and OBP2B can bind reversibly to odorants [23].

The *AMBP* gene encodes α_1_-microglobulin/bikunin precursor protein; α_1_-MG (A1M) is the lipocalin—derived from proteolytic cleavage of AMBP [24]. A1M is secreted into plasma, where it can exist free, or bound, to immunoglobulin-A or albumin. Although the molecular weight of A1M is 27.0 kDa, it is freely filtered through the glomerulus and reabsorbed by proximal tubular cells [25]; for this reason, A1M is a biomarker of proteinuria, i.e., increased levels in urine indicate a defect in proximal tubules. A1M is considered to be a major factor for progressive impairment of renal function, as well as for early diagnosis of acute allograft rejection [13, 24, 26]. Recent studies have shown A1M to be expressed in rat retinal explants and to have oxygen radical-scavenging and reductase properties; these findings suggest that A1M might protect against oxidative stress and possibly be involved in the response to retinal detachment [27, 28].

Other members of the LCN family include apolipoproteins D (APOD) and M (APOM)—which interestingly exhibit structural similarities to LCNs rather than to other apolipoproteins. APOD is an atypical apolipoprotein, because it is highly expressed in mammalian tissues such as liver, kidney, and central nervous system. APOD is a component of HDL cholesterol. Recent studies have shown that abnormal APOD expression is associated with altered lipid metabolism; three distinct missense mutations (Phe36Val, Tyr108Cys, and Thr158Lys) in African populations link APOD with metabolic syndrome [29]. A recent study showed that APOM, which resides in the plasma HDL fraction, acts as a chaperone for sphingosine-1-phosphate (S1P) and facilitates interaction between S1P and plasma HDL, thereby exhibiting a vasculoprotective effect [30].

The protein encoded by the complement C8 gamma chain gene (*C8G*) is one of the three subunits present in complement component 8 (C8). It is an oligomeric protein composed of three non-identical sub-units (α, 64-kDa; β, 64-kDa; γ, 22-kDa); the gamma chain is the only one that belongs to the lipocalin family [31]. C8 is part of the membrane-attack complex (MAC) that participates in irreversible association of the complement proteins C5b, C6, C7, and C9 to form a cytolytic complex that inserts into, and directly lyses, microbes [32]. Activation of complement triggers the assembly of MAC, which is then deployed to kill a wide range of Gram-negative bacteria [33]. Two functionally distinct C8-deficiency states have been identified: the first reflects a lack of the alpha and gamma chains and has been reported in Afro-Caribbean, Hispanic, and Japanese populations; the second results from lack of the beta chain and is found mainly in Caucasians [34, 35]. Deficiency of C8 complement is a very rare primary immunodeficiency associated with invasive and recurrent infections by *Neisseria meningitidis* [32, 36, 37].

Orosomucoids (ORM1 and ORM2), α_1_-acid glycoproteins (trivial name AGPs), belong to the subfamily of immunocalins. ORM1 is an acute phase protein secreted by hepatocytes in response to inflammation, with its expression being regulated by pro-inflammatory cytokines such as IL1 and IL6, the chemokine IL8, and glucocorticoids [38]. ORM1 and ORM2 are polymorphic proteins—commonly referred to as ORM/AGP with four variants in humans: AGP F1; AGP F2; AGP S, encoded by the *ORM1* gene; and AGP A, encoded by the *ORM2* gene [39]. AGPs are important members of the lipocalin family, because their capacity to bind to basic drugs can affect plasma free drug concentrations, playing a key role in a drug’s volume of distribution, metabolism, and therapeutic effect [40]. The ORM1 and ORM2 proteins have been recently identified as predictive urinary biomarkers for rheumatoid arthritis [41]. In addition, they are predictive markers for systemic lupus [42] and chronic inflammation [43].

The progestagen-associated endometrial protein (PAEP) is a secreted immunosuppressive glycoprotein (28 kDa), also termed glycodelin, i.e., one of the immunocalins. Studies have shown that PAEP downregulation can lead to abortion during the first trimester—due to increased activation of the immune system [44, 45]. In addition, PAEP has been found expressed in many tumors (e.g., gynecological malignancies, lung cancer, and melanoma) [46–48].

The protein encoded by the prostaglandin D_2_ synthase (*PTGDS*) gene is a glutathione-independent prostaglandin synthase (PTGDS). PTGDS is involved in the arachidonic acid cascade, converting prostaglandin H_2_ to prostaglandin D_2_ (PGD2), and is preferentially expressed in brain [49]. Increased PTGDS expression has been shown in patients having attention deficit hyperactivity disorder, compared with patients having bipolar disorder [50]. Another study suggests that dysregulated *PTGDS* mRNA expression is associated with rapid-cycling bipolar depression [49]. Enhanced PTGDS expression has also been associated with various malignancies [51–55].

Plasma retinol-binding protein 4 (RBP4) is a 21-kDa transporter of all-*trans*-retinol and belongs to the lipocalin family [56, 57]. RBP4 circulates in plasma as a moderately tight 1:1 M complex with vitamin A. RBP4 is secreted mainly by hepatocytes and also by adipose tissue [58]. In humans, increased circulating RBP4 levels have been correlated with obesity [59], insulin resistance, and type-2 diabetes [60, 61]. Insulin resistance has been long considered to play a key role in the development of non-alcoholic fatty liver disease (NAFLD) [62]—which is associated with altered RBP4 levels. Information in the literature on this association, however, is controversial. Several studies have reported significantly increased RBP4 levels in patients with NAFLD [63–66], whereas other studies have shown no difference on RBP4 levels between control and NAFLD groups [67, 68].

There is limited information in the literature regarding human LCN6, LCN8, LCN9, LCN10, LCN12, or LCN15.

## Lipocalin family in mice

**Fig. 2:**
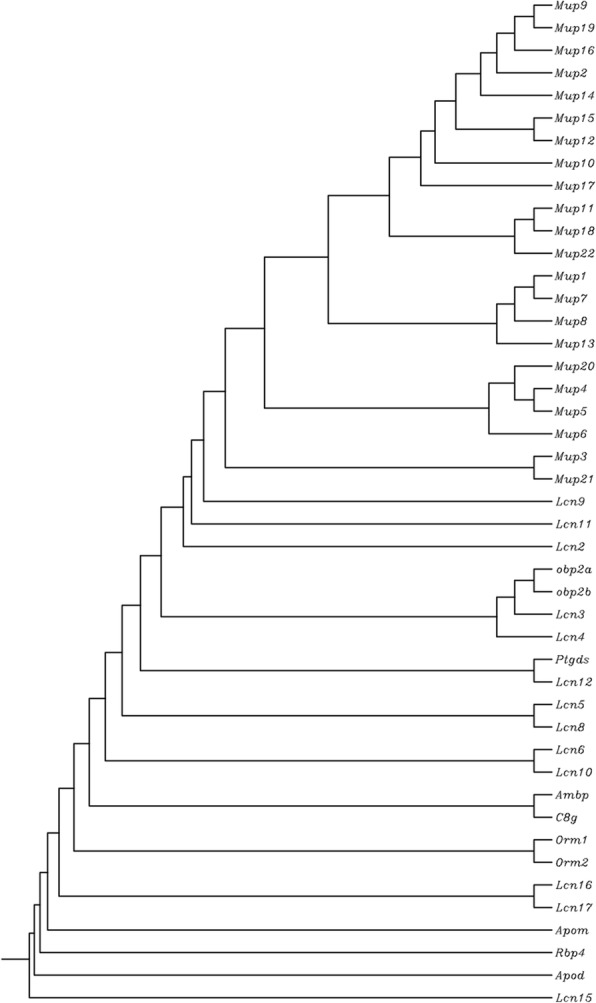
Dendrogram of mouse *Lcn* and *Mup* proteins. Although the names listed are the official mouse gene symbols [http://www.informatics.jax.org/], this dendrogram is based on the alignment of proteins (listed in Tables 1 and 2), using multiple sequence alignment by CLUSTALW (http://www.genome.jp/tools/clustalw/)

Lipocalins have been extensively studied in the mouse. Forty-five proteins belong to this family in mice (Table 1), which also includes major urinary proteins (MUPs) as members of this family (Fig. 2). All of the *LCN* genes are expressed in both humans and mice (Table 1), with the only exception of *LCN1*, which is found only in human—whereas *Lcn3*, *Lcn4*, *Lcn5*, *Lcn11*, *Lcn16*, *Lcn17*, and all the functional *Mup* genes are found in mouse but not human.

MUPs are intriguing small proteins (19–21 kDa) found in mouse urine; in rats, MUPs are known as α_2u_-globulins [69–72]. (For the purpose of this review, MUPs will refer to major urinary proteins in both mouse and rat.) While the presence of proteinuria is considered in humans to be a pathological renal condition, this is not the case for mice or rats [70]. Under physiological conditions, rodents excrete substantial levels of protein in urine, with MUPs accounting for > 90% of total protein content [70, 73, 74], playing a key role in chemo-signaling between animals to coordinate social behavior [75]. MUPs represent highly homologous proteoforms that control the release of volatile pheromones for urinary scent marks by transporting them into the vomeronasal organ (VNO) [76, 77]. MUPs bind pheromones within the hydrophobic calyx of the protein structure where hydrophobic binding sites exist for small lipophilic ligands. The affinity of each MUP for specific ligands varies, according to its subtype [70, 78], and depends on the amino-acid sequence in the binding domain [79, 80]. MUP affinity is most affected by polymorphisms that influence amino acids on the luminal surface of the ligand-binding domain (pocket)—rather than on the protein surface where most sequence differences are observed [73]. In addition, MUPs may act as direct stimulants of pheromone receptors [81].

MUPs are primarily synthesized in post-pubescent mouse liver in response to various hormones such as testosterone, growth hormone, thyroxine, insulin, and glucocorticoids [82, 83]. MUP synthesis is sex-dependent—resulting in (three- to fourfold) higher protein concentrations in post-pubescent males than female mice [84].

MUP expression is stimulated by androgens and leads to higher expression levels in adult males than in females, as well as immature males [70, 85]. Because their expression is stimulated by androgens, MUP synthesis is gender-dependent with higher (three- to fourfold) protein levels occurring in adult males than in females or immature males [70, 85]. For example, in C57/BL6 mice, MUPs represent 3.5–4% of total protein synthesized in male liver, but only 0.6–0.9% in female liver [86]. *Mup* mRNA is also expressed in a number of secretory tissues—such as nasal tissue, mammary, salivary, submaxillary, and lacrimal glands [87, 88]—as well as skeletal muscle, kidney, brain, spleen, heart, epididymal adipocytes, and brown adipose tissue [89–91]. MUP synthesis is initiated in response to different hormonal signals during various developmental stages; for example, liver synthesis of MUPs begins at onset of puberty and on through adulthood [92], whereas MUP synthesis in lacrimal gland starts 1 to 2 weeks before onset of puberty and continues into adulthood [83]. In addition, the specific *Mup* mRNA subtype produced varies from tissue to tissue [93] (Table 2).

**Table 2:** Mouse tissues known to express *Mup* mRNA [93]

Mup mRNA	Tissue
*Mup1*	Liver
*Mup2*	Mammary gland, liver
*Mup3*	Liver
*Mup4*	Parotid gland, lacrimal gland, nasal expression
*Mup5*	Submandibular gland, sublingual gland, lacrimal gland
*Mup6*	Parotid gland

## The *MUP* gene cluster in mouse and human genomes

Interestingly, the mouse *Mup* gene cluster (22 protein-coding genes; Table 3) can be divided into two subgroups. The first group (*Mup3*, *Mup4*, *Mup5*, *Mup6*, *Mup20*, and *Mup21*) is slightly older (Fig. 2) and contains a more divergent class of genes. The second group comprises the remaining 16 *Mup* genes, which share almost 99% sequence identity [75, 94]. The predicted gene, previously designated Gm21320 (“gene model 21320”), has now been renamed *Mup22*, cf. [http://www.informatics.jax.org/].

**Table 3:** List of all mouse *Mup* genes [http://www.informatics.jax.org/], with official gene symbols, aliases, chromosomal locations, isoforms, National Center for Biotechnology Information (NCBI) RefSeq mRNA accession numbers, NCBI RefSeq protein accession numbers, and total number of amino acids (# of AAs) [information retrieved and confirmed from https://www.ncbi.nlm.nih.gov/genome.]

Gene symbol	Aliases	Chromosome	Isoforms	Ref seq mRNA number	Ref seq protein number	Full protein name	No. of AAs
*Mup1*	Major urinary protein-1	Mup7; Up-1; Ltn-1; Mup-1; Mup-a; Mup10; Lvtn-1	4	MUP 1 isoform b precursor	NM_001163010.1	NP_001156482.1	121
*Mup2*	Major urinary protein-2	Mup4; Mup-2; AA589603	4	MUP 2 isoform 1 precursor	NM_001045550.2	NP_001039015.1	180
			4	MUP 2 isoform 2	NM_001286096.1	NP_001273025.1	119
			4	MUP 2 isoform 1 precursor	NM_008647.4	NP_032673.3	180
*Mup3*	Major urinary protein-3	MUP15; Mup-3; Mup25; MUPIII	4	MUP 3 precursor	NM_001039544.1	NP_001034633.1	184
*Mup4*	Major urinary protein-4	Mup1; Mup-4	4	MUP 4 precursor	NM_008648.1	NP_032674.1	178
*Mup5*	Major urinary protein-5	Mup18	4	MUP 5 precursor	NM_008649.2	NP_032675.2	180
*Mup6*	Major urinary protein-6	Mup2; Gm12544; OTTMUSG00000007423	4	MUP (Mup)-like precursor	NM_001081285.1	NP_001074754.1	179
*Mup7*	Major urinary protein-7	Mup3; Gm12546; OTTMUSG00000007428	4	MUP 7 precursor	NM_001134675.1	NP_001128147.1	235
*Mup8*	Major urinary protein-8	Mup5; Gm12809; OTTMUSG00000008509	4	MUP 8 precursor	NM_001134676.1	NP_001128148.1	235
*Mup9*	Major urinary protein-9	Mup2; Mup6; Gm14076; OTTMUSG00000015595	4	MUP 2-like precursor	NM_001281979.1	NP_001268908.1	180
*Mup10*	Major urinary protein-10	Mup8; 2610016E04Rik	4	MUP 10 precursor	NM_001122647.1	NP_001116119.1	180
*Mup11*	Major urinary protein-11	Gm12549; OTTMUSG00000007431	4	MUP 11 precursor	NM_001164526.1	NP_001157998.1	181
*Mup12*	Major urinary protein-12	Gm2024	4	MUP 12 precursor	NM_001199995.1	NP_001186924.1	180
*Mup13*	Major urinary protein-13	Mup11; Gm13513; OTTMUSG00000012492	4	MUP 13 precursor	NM_001134674.1	NP_001128146.1	235
*Mup14*	Major urinary protein-14	Mup12; Gm13514; OTTMUSG00000012493	4	MUP 14 precursor	NM_001199999.1	NP_001186928.1	180
*Mup15*	Major urinary protein-15	Mup13; Gm2068	4	MUP 15 precursor	NM_001200004.1	NP_001186933.1	180
*Mup16*	Major urinary protein-16		4	MUP 16 precursor	NM_001199936.1	NP_001186865.1	180
*Mup17*	Major urinary protein-17	MUP 17; Gm12557; OTTMUSG00000007480	4	MUP 17 precursor	NM_001200006.1	NP_001186935.1	180
*Mup18*	Major urinary protein-18	Mup6	4	MUP 6 precursor	NM_001199333.1	NP_001186262.1	181
*Mup19*	Major urinary protein-19	Mup8; Mup11; Mup14; Mup17; Gm12552; 100039247; OTTMUSG00000007472	4	MUP 11 and 8 precursor	NM_001135127.2	NP_001128599.1	180
*Mup20*	Major urinary protein-20	Mup24; darcin; Gm12560; OTTMUSG00000007485	4	MUP 20 precursor	NM_001012323.1	NP_001012323.1	181
*Mup21*	Major urinary protein-21	Mup; Mup26; Gm11208; bM64F17.1; bM64F17.4; OTTMUSG00000000231	4	MUP 26 precursor	NM_001009550.2	NP_001009550.1	181
*Mup22*	Major urinary protein-22	Gm21320	4	MUP 2-like isoform X1	XM_003688783.3	XP_003688831.1	181

**Fig. 3:**
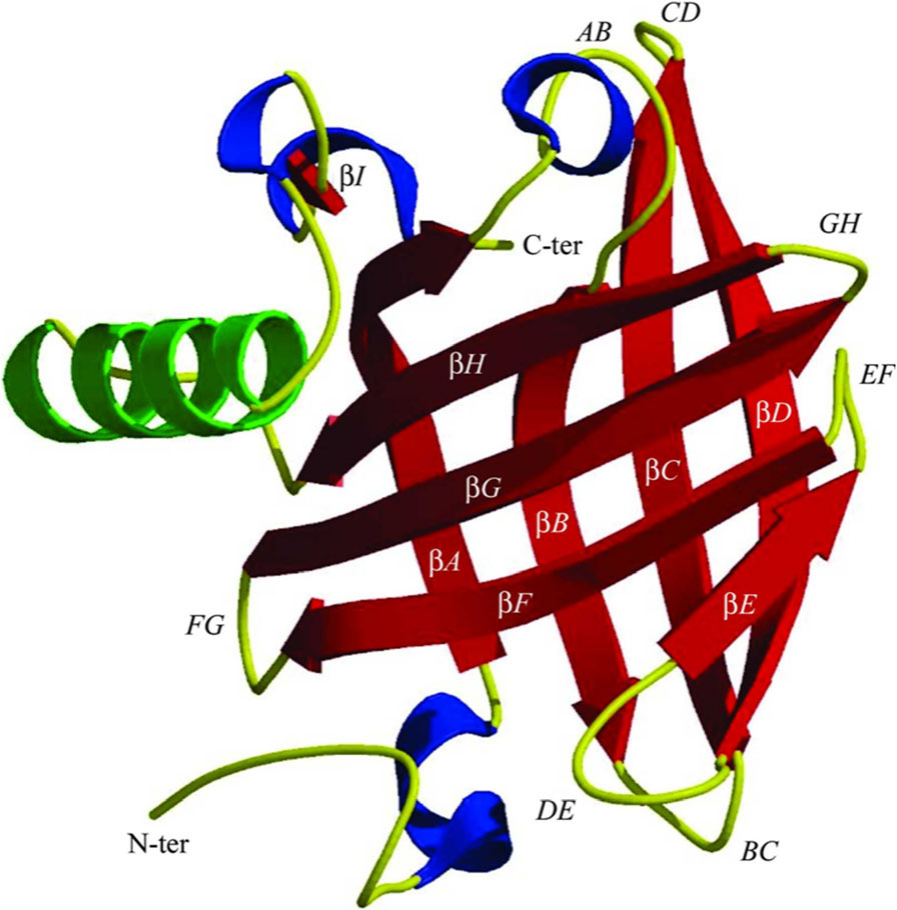
Structure of prototypical mouse urinary protein. The crystal structure consists of eight β-strands, forming a calyx-shaped barrel (*red*); this encloses an internal ligand-binding site. There are also an α-helix (*green*) and four 3_10_-helices (*blue*); the hydrophobic pocket is located inside the barrel. *AB*, *BC*, *CD*, *DE*, *EF*, *FG*, *GH*, *HI*, *AND* β*I* denote the amino-acid segments between the β-strands (*This diagram taken from Ref.* [95])

**Fig. 4:**
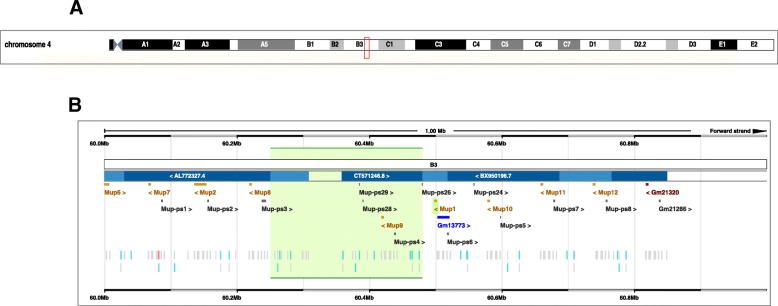
Chromosomal location of mouse *Mup* genes and pseudogenes. **a** The *Mup* cluster region, located at 60,498,012 Mb to 60,501,960 Mb (*red vertical rectangle*). Taken from the Ensembl genome browser. **b** The Chr 4 region (in greater detail)—showing ten of the 22 *Mup* genes (Gm21320 is *Mup22*) in the *Mup* cluster and 12 of the 29 *Mup-ps* pseudogenes

As members of the LCN family, MUPs exhibit conservation in the common three-dimensional structure of the protein family, i.e., a central area pocket formed by eight hydrophobic β-strand domains that form a barrel (Fig. 3) [81, 95]. This structure enables the MUPs to serve as carrier proteins for small lipophilic molecules such as pheromones and other chemical signals [78, 81]. All 22 mouse *Mup* protein-coding genes are located in a cluster (the *Mup* locus) on Chr 4 (Fig. 4 a, b) [96]. There are also 29 *Mup-ps* pseudogenes in the Chr 4 Mup cluster (intriguingly, the one remaining pseudogene, *Mup-ps22*, is located on Chr 11).

An “evolutionary bloom” is defined when one sees a recent, phylogenetically independent proliferation of close paralogs or lineage-specific gene family expansion [97]. Examples of this phenomenon have been extensively studied in the large and diverse cytochrome P450 superfamily [97]. For example, the koala’s ability to detoxify eucalyptus leaves appears to be due to an evolutionary bloom within a cytochrome P450 gene group; the koala’s *CYP2C* subfamily was found to comprise 31 putative protein-coding functional enzymes, compared to 15 *Cyp2c* genes in mouse and just four *CYP2C* genes in human [98]. Another example is the mouse *Scgb* gene superfamily—which includes a number of encoded androgen-binding proteins involved in mate selection [99]; this is fascinating, because the *Mup* cluster (described herein) also encodes proteins involved in mate selection. It has been suggested that these evolutionary blooms might represent simply a stochastic process [97]. However, it is more likely these blooms are the result of environmental pressures needed for the organism to survive (i.e., find food, avoid predators, and reproduce) at a particular moment in evolutionary time.

*Mup* gene polymorphisms in rat and mouse have shown significant differences. Yet, such differences have not been seen in genomes of other mammalian species for which whole-genome sequences have been explored [75]. Although the amino acid sequence of MUP homologs between rat and mouse is ~ 65%, there is a characteristic six amino-acid consensus sequence (Glu-Glu-Ala-Ser-Ser-Thr) that remains highly conserved between these two species [100]. In general, species differences in MUP proteins appear to be mainly due to glycosylated MUP amino-acid residues that occur in rats, but not mice [100]. Most other mammalian species (e.g., dog, baboon, gorilla, and chimpanzee) have only one functional protein-coding *MUP* gene, except for horse that has three functional *MUP* genes [75].

The human *MUP*-related gene is a pseudogene (*MUPP*, located at Chr 9q32). Using the UCSC genome browser [https://genome.ucsc.edu/], one can visualize that human Chr 9q32 is syntenic to mouse Chr 4 at 60,498,012 Mb to 60,501,960 Mb, where the *Mup* cluster of 22 *Mup* genes is located; in fact, the human *ZFP37* and mouse *Zfp37* gene flank the “MUP region” in both human and mouse, respectively. The human *MUPP* locus exhibits a high degree of sequence similarity to mouse *Mup* functional genes but contains coding-sequence disruptions that prevent the gene product from being formed [101]. The human *MUPP* shows a G > A transition (relative to the chimpanzee *MUP* sequence) that disrupts a splice-donor site [75]; this is interesting because this G > A mutation has not been observed in mammals other than humans [101]. The human *MUPP* pseudogene sequence is most similar to the mouse *Mup-ps4* pseudogene [75].

One of the main functions of MUP proteins is to promote aggressive behavior through binding to vomeronasal pheromone receptors (V2Rs) in the accessory olfactory neural pathway. Even though there is a co-expansion of MUPs and V2Rs in mouse, rat, and opossum—all human V2R receptors have become inactive, possibly leading to the pseudogenization of the single human *MUP* gene [102, 103]. In other words, the absence of the specific V2R removed the selection pressure for a functional MUP ligand.

## Parallel expansions of *Mup* clusters

The last common ancestor of rat and mouse had either a single, or a small number of, *Mup* genes [75]. By determining the extent of *Mup* gene expansions across non-rodent lineages, Logan and colleagues were able to identify orthologs of the *Slc46a2* and *Zfp37* genes (and the contiguous genomic sequence spanning the interval between these two genes) in nine additional placental mammals [75]. Whereas C57BL/6J mice have a cluster of 22 distinct *Mup* genes on Chr 4 and rats have nine distinct *Mup* genes, mammalian species such as dog, pig, baboon, chimpanzee, bush baby, and orangutan—each has a single *Mup* gene (with no evidence of additional pseudogenes). By contrast, the human genome has only the one pseudogene.

**Fig. 5:**
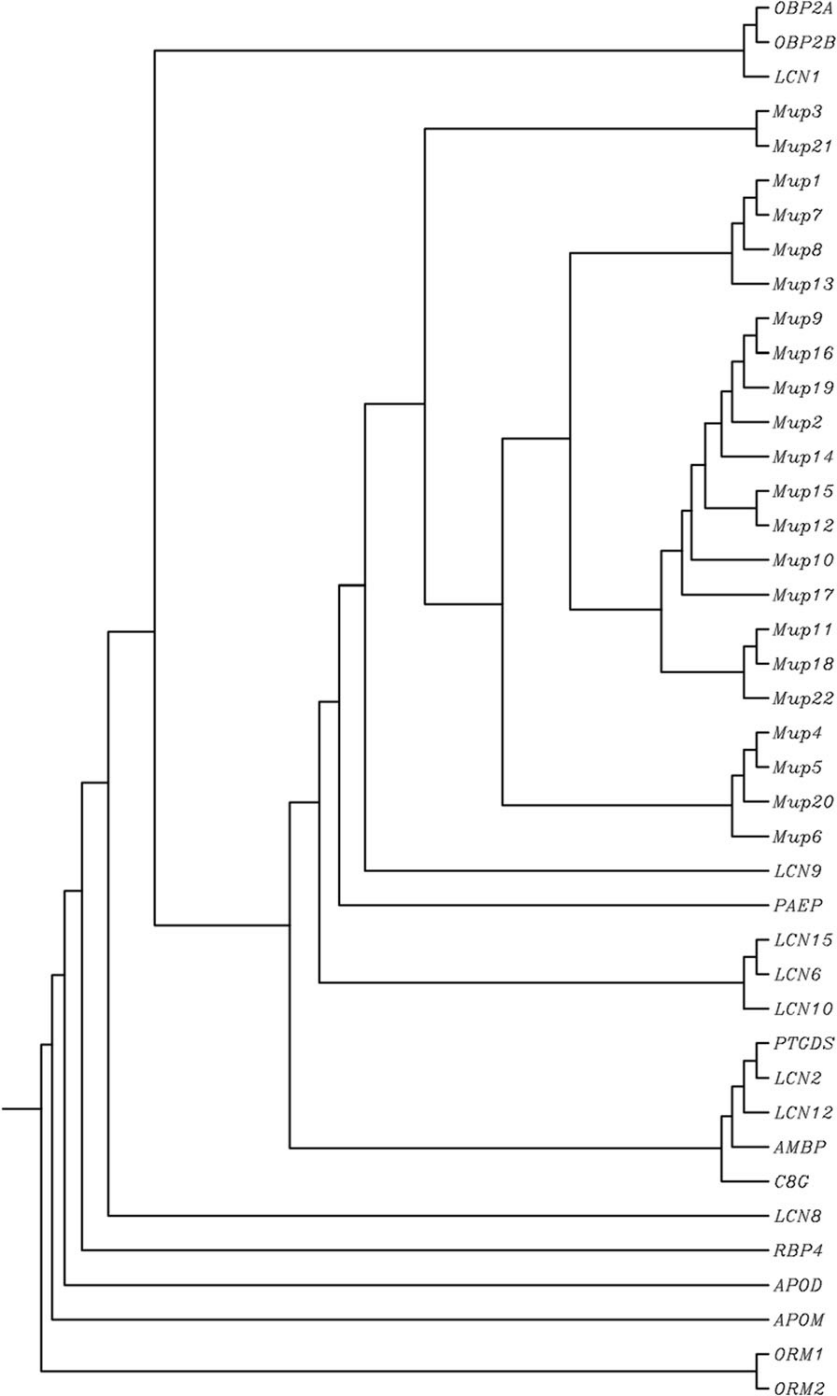
Dendrogram of human LCNs and mouse MUPs, combined. Although the names listed are the official human gene symbols and mouse *Mup* gene symbols, this dendrogram was based on the alignment of proteins (listed in Tables 1 and 3) using multiple sequence alignment by CLUSTALW (http://www.genome.jp/tools/clustalw/). Note that the human *LCN9* gene is evolutionarily closest to the mouse *Mup* cluster in this dendrogram

A neighbor-joining dendrogram of human LCN and mouse MUP proteins is illustrated in Fig. 5; subfamilies can be distinguished based on evolutionary divergence. Note that all mouse MUPs are clustered into a subgroup near the top of the dendrogram, whereas the human *LCNs* are split into several different branches—due to the high degree of divergence of LCN proteins. The mouse *Mup* cluster divergence is most closely associated with human LCN9 and PAEP (Fig. 5). Note that the evolutionarily oldest human *LCN* genes include *ORM1*, *ORM2*, *APOM*, *APOD*, *RBP4*, and *LCN8*.

## Functions of MUPs in mice

### MUPs and chemical communication

Due to their influence on pheromones, MUPs appear to be involved in regulating transmission of social signals—such as identity, territorial marking, and mate choice [104–106]. Most pheromones are small volatile molecules that influence aggression, mating, feeding, and territorial behavior within the same species [103, 107]. Mice use pheromones as cues to regulate social behaviors. Neurons that detect pheromones reside in at least two separate organs within the nasal cavity: the vomeronasal organ (VNO) and the main olfactory epithelium (MOE). Each pheromone molecule is thought to activate a dedicated subset of these sensory neurons—similar to the manner in which odorants are received by dedicated subsets of mammalian olfactory receptors. However, the identity of the responding neurons that regulate specific social behaviors remains largely unknown.

Pheromones have a short half-life, which can be prolonged by binding to the characteristic barrel pocket, in the MUP protein. In addition, gradual release of a pheromone from a MUP protein allows the half-life of these airborne odor signals to be extended, e.g., to be used as mammalian scent marks [108, 109].

MUPs are also linked to reproductive success in males [110] and to social behavior, by adjusting the animal’s odor profile in response to different stimuli. This function underscores how social environment plays an important role in MUP production in both male and female mice. For example, MUP synthesis is upregulated in a male mouse housed with a female, but downregulated when a male mouse is housed with other males only [111]. Perhaps related to this, MUPs can be predictive of the onset of aggressive and dispersal behavior among male mice [103].

In addition to serving as pheromone carriers, MUPs can function as pheromones themselves. They facilitate chemical information exchange to convey specific information (e.g., gender, social and reproductive status) between animals [70]. Recent research has revealed that MUPs also act as kairomones, causing a fear reaction in response to predators [112]. For example, the rat kairomone that triggers defensive behavior in mice is encoded by the *Mup13* gene [112].

Members of the MUP family are known to be involved in intraspecies interplay, especially male-male aggression in mice [103]. Female mice are attracted to urine-borne male pheromones. MUP20, for example, has been shown to be rewarding and attractive to female mice. MUPs excreted by male mice can also influence reproductive behavior and promote female attraction [103, 113–115]. The molecular mechanism promoting spontaneous ovulation involves direct stimulation of VNO nerves by four residues on the NH_2_-terminus of MUP proteins [116]. MUPs may also be involved in mediating individual recognition and inbreeding avoidance [117, 118].

### MUPs and metabolism

MUPs also appear to be involved in energy metabolism—actions reminiscent of the lipocalins that have been implicated clinically in lipid disorders and metabolic syndrome caused by obesity and type-2 diabetes [119, 120]. For example, mouse MUP1 regulates systemic glucose metabolism by modulating the hepatic gluconeogenic and/or lipogenic programs [121–123]. Caloric restriction dramatically reduces MUP1 expression in mouse liver [124, 125] and appears to decrease MUP4 and MUP5 expression, as well [124, 126].

Decreased hepatic MUP1 levels have been linked to obesity and type-2 diabetes in mice with either genetic (leptin receptor-deficient *db/db*) or dietary fat-induced obesity [121, 127]. Similar decreases in MUP1 are found in extrahepatic organs—such as adipose tissue and the hypothalamus—after caloric restriction [128, 129]. Furthermore, MUP1 was also found to lower blood glucose levels by inhibiting expression of phosphoenolpyruvate carboxykinase and glucose-6-phosphatase, two rate-limiting enzymes for gluconeogenesis [127]. These studies suggest that mouse MUP1, and possibly other MUP family members, are playing key roles in energy metabolism and potentially contributing to the development of metabolic diseases such as type-2 diabetes.

## Human *LCN*-like genes and their mouse orthologs

We have listed the human *LCN*-related genes in Table 1 and mouse *Lcn*-like genes plus the *Mup* cluster genes in Table 3. What are the percent similarities of proteins—if one compares human *LCN*-like genes with their mouse orthologs? Among the 19 *LCN*-like genes in the human genome (Table 4), 17 have mouse orthologs, whereas human *LCN1* and *PAEP* do not. Within the *LCN* cluster, *LCN6* and *LCN8* exhibit the highest percent similarity: 74 and 70%, respectively. Among all 19 *LCN*-like genes, *RBP4* and *APOM* reveal the highest percent similarity (86% and 81%, respectively); *LCN15* and *OBP2A* display the lowest percent similarity (39%) to their mouse orthologs.

**Table 4:** List of the 19 *LCN*-like genes in the human genome and their similarity to their mouse orthologs, expressed as percent identity (%)

Gene	Similarity to mouse (percent identity (%))
*LCN1*	NA
*LCN2*	62
*LCN6*	74
*LCN8*	70
*LCN9*	54
*LCN10*	64
*LCN12*	56
*LCN15*	39
*OBP2A*	39
*OBP2B*	50
*AMBP*	77
*APOD*	74
*APOM*	81
*C8G*	76
*ORM1*	49
*ORM2*	44
*PAEP*	NA
*PTGDS*	72
*RBP4*	86

Human and mouse ancestors are estimated to have diverged from one another ~ 80 million years ago. Table 4 confirms the relatively rapid rate of evolutionary divergence by the *LCN*-like genes, which is consistent with their function of requiring evolutionarily quick adaptation to changing environments; this is similar to, e.g., beta-defensin (*DEFB*) genes, which number almost four dozen in the human genome and encode broad-spectrum antimicrobial cationic peptides [130]. Out of 19 *LCN*-like genes, the appearance of two novel human genes (*LCN1* and *PAEP*) during the past ~ 80 million years is further evidence of an enhanced evolutionary rate for this gene superfamily.

Rapid rates of evolutionary divergence stand in sharp contrast to, e.g., highly conserved transcription factors, whose human-mouse orthologs are generally > 95% similar in protein sequence. In fact, assaying for complementation of lethal growth defects in yeast, almost half (47%) of the yeast genes could be successfully humanized [131], and the yeast-human divergence occurred well over one billion years ago.

## Conclusions

Lipocalins (LCNs) are members of a family of evolutionarily conserved small proteins that possess a binding pocket. The LCN proteins (18–40 kDa) are encoded by 19 human *LCN*-related genes and 45 mouse *Lcn*-related genes. LCN proteins are expressed in numerous tissues and play important roles in physiological processes by transporting molecules in plasma and other body fluids. In humans, LCNs are extensively used clinically as biochemical markers in various diseases—such as diabetic renal disease, systemic lupus erythematosus, and chronic inflammation.

In mice, major urinary proteins (MUPs) are also members of the lipocalin family. The *Mup* cluster of 22 functional protein-coding *Mup* genes (plus 29 of 30 *Mup-ps* pseudogenes) is confined to mouse Chr 4 and represents an “evolutionary bloom,” because only one or a few *MUP* genes are functional in other mammals. In fact, no functional *MUP* gene exists in the human genome—although a human *MUPP* pseudogene located at Chr 9q32 is syntenic to the *Mup* cluster on Chr 4.

The MUP protein structure contains a conserved “barrel” formed by the eight β-chains having the characteristic central hydrophobic pocket binding-site. Mouse MUP proteins are expressed mainly in the liver, secreted into the bloodstream, and excreted by the kidney. MUPs are involved in the communication of information in urine-derived scent marks and can also serve as pheromones themselves. Circulating MUPs may also contribute to regulation of nutrient metabolism—possibly by suppressing hepatic gluconeogenic and lipid metabolism. However, it still remains unclear how MUPs, especially mouse MUP1, regulate energy metabolism and the gluconeogenic pathway. Further studies will be needed to shed light on these mechanisms.

## References

[1] Akerstrom B, Flower DR, Salier JP (2000). Lipocalins: unity in diversity. Biochim Biophys Acta..

[2] di Masi A (2016). Human plasma lipocalins and serum albumin: plasma alternative carriers?. J Control Release..

[3] Flower DR, North AC, Attwood TK (1993). Structure and sequence relationships in the lipocalins and related proteins. Protein Sci..

[4] Flower DR (1996). The lipocalin protein family: structure and function. Biochem J..

[5] Schiefner A, Skerra A (2015). The menagerie of human lipocalins: a natural protein scaffold for molecular recognition of physiological compounds. Acc Chem Res..

[6] Bocskei Z (1992). Pheromone binding to two rodent urinary proteins revealed by X-ray crystallography. Nature..

[7] Ganfornina MD (2000). A phylogenetic analysis of the lipocalin protein family. Mol Biol Evol..

[8] Sanchez D, Ganfornina Álvarez MD, Gutierrez G, Gauthier-Jauneau AC, Risler JL, Salier JP. In: Akerstrom B, editor. Lipocalin genes and their evolutionary history, in (Molecular Biology Intelligence Unit: Lipocalins): Landes Bioscience, Inc; 2006. p. 1–12.

[9] Akerstrom B (2000). alpha(1)-Microglobulin: a yellow-brown lipocalin. Biochim Biophys Acta..

[10] Alok A, Mukhopadhyay D, Karande AA (2009). Glycodelin A, an immunomodulatory protein in the endometrium, inhibits proliferation and induces apoptosis in monocytic cells. Int J Biochem Cell Biol..

[11] Logdberg L, Wester L (2000). Immunocalins: a lipocalin subfamily that modulates immune and inflammatory responses. Biochim Biophys Acta..

[12] Wang JC (2017). Detection of low-abundance biomarker lipocalin 1 for diabetic retinopathy using optoelectrokinetic bead-based immunosensing. Biosens Bioelectron..

[13] Bazzi C (2001). Urinary excretion of IgG and alpha(1)-microglobulin predicts clinical course better than extent of proteinuria in membranous nephropathy. Am J Kidney Dis..

[14] Karnati R, Laurie DE, Laurie GW (2013). Lacritin and the tear proteome as natural replacement therapy for dry eye. Exp Eye Res..

[15] Srinivasan S (2012). iTRAQ quantitative proteomics in the analysis of tears in dry eye patients. Invest Ophthalmol Vis Sci..

[16] Glasgow BJ, Gasymov OK (2011). Focus on molecules: tear lipocalin. Exp Eye Res..

[17] Moschen AR (2017). Lipocalin-2: a master mediator of intestinal and metabolic inflammation. Trends Endocrinol Metab..

[18] Nairz M (2015). Lipocalin-2 ensures host defense against Salmonella typhimurium by controlling macrophage iron homeostasis and immune response. Eur J Immunol..

[19] Li L (2009). Serum retinol-binding protein 4 is associated with insulin secretion in Chinese people with normal glucose tolerance. J Diabetes..

[20] Singh RG (2017). Role of human lipocalin proteins in abdominal obesity after acute pancreatitis. Peptides..

[21] Wang E (2017). Overexpression of exogenous kidney-specific Ngal attenuates progressive cyst development and prolongs lifespan in a murine model of polycystic kidney disease. Kidney Int..

[22] Lacazette E, Gachon AM (2000). Pitiot G. A novel human odorant-binding protein gene family resulting from genomic duplicons at 9q34: differential expression in the oral and genital spheres. Hum Mol Genet..

[23] Tegoni M (2000). Mammalian odorant binding proteins. Biochim Biophys Acta..

[24] Stubendorff B (2014). Urine protein profiling identified alpha-1-microglobulin and haptoglobin as biomarkers for early diagnosis of acute allograft rejection following kidney transplantation. World J Urol..

[25] Hong CY (2003). Urinary alpha1-microglobulin as a marker of nephropathy in type-2 diabetic Asian subjects in Singapore. Diabetes Care..

[26] Amer H (2013). Urine high and low molecular weight proteins one-year post-kidney transplant: relationship to histology and graft survival. Am J Transplant..

[27] Cederlund M (2013). Vitreous levels of oxidative stress biomarkers and the radical-scavenger alpha1-microglobulin/A1M in human rhegmatogenous retinal detachment. Graefes Arch Clin Exp Ophthalmol..

[28] Akerstrom B (2017). The role of mitochondria, oxidative stress, and the radical-binding protein A1M in cultured porcine retina. Curr Eye Res..

[29] Desai PP (2002). Genetic variation in the apolipoprotein D gene among African blacks and its significance in lipid metabolism. Atherosclerosis..

[30] Christoffersen C (2011). Endothelium-protective sphingosine-1-phosphate provided by HDL-associated apolipoprotein M. Proc Natl Acad Sci U S A..

[31] Chiswell B (2007). Structural features of the ligand binding site on human complement protein C8gamma: a member of the lipocalin family. Biochim Biophys Acta..

[32] Serna M (2016). Structural basis of complement membrane attack complex formation. Nat Commun..

[33] Figueroa JE, Densen P (1991). Infectious diseases associated with complement deficiencies. Clin Microbiol Rev..

[34] Kotnik V (1997). Molecular, genetic, and functional analysis of homozygous C8 beta-chain deficiency in two siblings. Immunopharmacology..

[35] Ross SC, Densen P (1984). Complement deficiency states and infection: epidemiology, pathogenesis and consequences of neisserial and other infections in an immune deficiency. Medicine (Baltimore)..

[36] Arnold DF (2009). A novel mutation in a patient with a deficiency of the eighth component of complement associated with recurrent meningococcal meningitis. J Clin Immunol..

[37] Dellepiane RM (2016). Invasive meningococcal disease in three siblings with hereditary deficiency of the 8(th) component of complement: evidence for the importance of an early diagnosis. Orphanet J Rare Dis..

[38] Ceciliani F, Pocacqua V (2007). The acute phase protein alpha1-acid glycoprotein: a model for altered glycosylation during diseases. Curr Protein Pept Sci..

[39] Nishi K (2011). Structural insights into differences in drug-binding selectivity between two forms of human alpha1-acid glycoprotein genetic variants, the A and F1*S forms. J Biol Chem..

[40] Ohbatake Y (2016). Elevated alpha1-acid glycoprotein in gastric cancer patients inhibits the anticancer effects of paclitaxel, effects restored by co-administration of erythromycin. Clin Exp Med..

[41] Gomes MB, Nogueira VG (2004). Acute-phase proteins and microalbuminuria among patients with type-2 diabetes. Diabetes Res Clin Pract..

[42] Watson L (2012). Urinary monocyte chemoattractant protein 1 and alpha 1 acid glycoprotein as biomarkers of renal disease activity in juvenile-onset systemic lupus erythematosus. Lupus..

[43] Singh R (2017). Urinary biomarkers as indicator of chronic inflammation and endothelial dysfunction in obese adolescents. BMC Obes..

[44] Toth B (2008). Glycodelin protein and mRNA is downregulated in human first trimester abortion and partially upregulated in mole pregnancy. J Histochem Cytochem..

[45] Xu S, Venge P (2000). Lipocalins as biochemical markers of disease. Biochim Biophys Acta..

[46] Ren S (2010). Functional characterization of the progestagen-associated endometrial protein gene in human melanoma. J Cell Mol Med..

[47] Scholz C (2012). Glycodelin A is a prognostic marker to predict poor outcome in advanced stage ovarian cancer patients. BMC Res Notes..

[48] Schneider MA (2015). Glycodelin: a new biomarker with immunomodulatory functions in non-mall cell lung cancer. Clin Cancer Res..

[49] Munkholm K (2014). Reduced mRNA expression of PTGDS in peripheral blood mononuclear cells of rapid-cycling bipolar disorder patients compared with healthy control subjects. Int J Neuropsychopharmacol..

[50] Marin-Mendez JJ (2012). Differential expression of prostaglandin D2 synthase (PTGDS) in patients with attention deficit-hyperactivity disorder and bipolar disorder. J Affect Disord..

[51] Kim GE, et al. Differentially expressed genes in matched normal, cancer, and lymph node metastases predict clinical outcomes in patients with breast cancer. Appl Immunohistochem Mol Morphol. 2018. 10.1097/PAI.0000000000000717 PMC7028469 32044879

[52] Zhang B, et al. PGD2/PTGDR2 signaling restricts the self-renewal and tumorigenesis of gastric cancer. Stem Cells. 2018. 10.1002/stem.2821 29604141

[53] Nault JC (2018). Argininosuccinate synthase 1 and periportal gene expression in sonic hedgehog hepatocellular adenomas. Hepatology..

[54] Davalieva K (2017). Comparative proteomics analysis of urine reveals down-regulation of acute phase response signaling and LXR/RXR activation pathways in prostate cancer. Proteomes..

[55] Omori K (2018). Lipocalin-type prostaglandin D synthase-derived PGD2 attenuates malignant properties of tumor endothelial cells. J Pathol..

[56] Zhou Z (2017). Circulating retinol binding protein 4 levels in nonalcoholic fatty liver disease: a systematic review and meta-analysis. Lipids Health Dis..

[57] Christou GA, Tselepis AD, Kiortsis DN (2012). The metabolic role of retinol binding protein 4: an update. Horm Metab Res..

[58] Newcomer ME, Ong DE (2000). Plasma retinol binding protein: structure and function of the prototypic lipocalin. Biochim Biophys Acta..

[59] Codoner-Franch P (2016). Association of RBP4 genetic variants with childhood obesity and cardiovascular risk factors. Pediatr Diabetes..

[60] Yang Q (2005). Serum retinol binding protein 4 contributes to insulin resistance in obesity and type-2 diabetes. Nature..

[61] Graham TE (2006). Retinol-binding protein 4 and insulin resistance in lean, obese, and diabetic subjects. N Engl J Med..

[62] Birkenfeld AL, Shulman GI (2014). Nonalcoholic fatty liver disease, hepatic insulin resistance, and type-2 diabetes. Hepatology..

[63] Seo JA (2008). Serum retinol-binding protein 4 levels are elevated in non-alcoholic fatty liver disease. Clin Endocrinol..

[64] Chen X (2017). Retinol binding protein-4 levels and non-alcoholic fatty liver disease: a community-based cross-sectional study. Sci Rep..

[65] Terra X (2013). Retinol binding protein-4 circulating levels were higher in nonalcoholic fatty liver disease vs. histologically normal liver from morbidly obese women. Obesity (Silver Spring)..

[66] Wu H (2008). Serum retinol binding protein 4 and nonalcoholic fatty liver disease in patients with type-2 diabetes mellitus. Diabetes Res Clin Pract..

[67] Cengiz C (2010). Serum retinol-binding protein 4 in patients with nonalcoholic fatty liver disease: does it have a significant impact on pathogenesis?. Eur J Gastroenterol Hepatol..

[68] Milner KL (2009). Adipocyte fatty acid binding protein levels relate to inflammation and fibrosis in nonalcoholic fatty liver disease. Hepatology..

[69] Beynon RJ, Hurst JL (2003). Multiple roles of major urinary proteins in the house mouse, Mus domesticus. Biochem Soc Trans..

[70] Gomez-Baena G (2014). The major urinary protein system in the rat. Biochem Soc Trans..

[71] Mudge JM (2008). Dynamic instability of the major urinary protein gene family revealed by genomic and phenotypic comparisons between C57 and 129 strain mice. Genome Biol..

[72] Thom MD, Stockley P, Jury F, Ollier WE, Beynon RJ, Hurst JL. The direct assessment of genetic heterozygosity through scent in the mouse. Curr Biol. 2008;(18)8:619–623. 10.1016/j.cub.2008.03.056 18424142

[73] Beynon RJ (2002). Polymorphism in major urinary proteins: molecular heterogeneity in a wild mouse population. J Chem Ecol..

[74] Krop EJ (2007). Recombinant major urinary proteins of the mouse in specific IgE and IgG testing. Int Arch Allergy Immunol..

[75] Logan DW, Marton TF, Stowers L (2008). Species specificity in major urinary proteins by parallel evolution. PLoS One..

[76] Yang H (2016). Mup-knockout mice generated through CRISPR/Cas9-mediated deletion for use in urinary protein analysis. Acta Biochim Biophys Sin (Shanghai)..

[77] Enk VM (2016). Regulation of highly homologous major urinary proteins in house mice quantified with label-free proteomic methods. Mol Biosyst..

[78] Rajkumar R (2010). Primary structural documentation of the major urinary protein of the Indian commensal rat (Rattus rattus) using a proteomic platform. Protein Pept Lett..

[79] Sharrow SD (2002). Pheromone binding by polymorphic mouse major urinary proteins. Protein Sci..

[80] Armstrong SD (2005). Structural and functional differences in isoforms of mouse major urinary proteins: a male-specific protein that preferentially binds a male pheromone. Biochem J..

[81] Timm DE (2001). Structural basis of pheromone binding to mouse major urinary protein (MUP-I). Protein Sci..

[82] Hastie ND, Held WA, Toole JJ (1979). Multiple genes coding for the androgen-regulated major urinary proteins of the mouse. Cell..

[83] Shaw PH, Held WA, Hastie ND (1983). The gene family for major urinary proteins: expression in several secretory tissues of the mouse. Cell..

[84] Beynon RJ, Hurst JL (2004). Urinary proteins and the modulation of chemical scents in mice and rats. Peptides..

[85] Kaur AW (2014). Murine pheromone proteins constitute a context-dependent combinatorial code governing multiple social behaviors. Cell..

[86] Berger FG, Szoka P (1981). Biosynthesis of the major urinary proteins in mouse liver: a biochemical genetic study. Biochem Genet..

[87] Utsumi M, Ohno K, Kawasaki Y, Tamura M, Kubo T, Tohyama M (1999). Expression of major urinary protein genes in the nasal glands associated with general olfaction. J Neurobiol..

[88] Guo J, Zhou A, Moss RL (1997). Urine and urine-derived compounds induce c-fos mRNA expression in accessory olfactory bulb. Neuroreport..

[89] Stopkova R (2007). Species-specific expression of major urinary proteins in the house mice (Mus musculus musculus and Mus musculus domesticus). J Chem Ecol..

[90] Hui X (2009). Major urinary protein-1 increases energy expenditure and improves glucose intolerance through enhancing mitochondrial function in skeletal muscle of diabetic mice. J Biol Chem..

[91] Stopková R (2016). Mouse lipocalins (MUP, OBP, LCN) are co-expressed in tissues involved in chemical communication. Front Ecol Evol..

[92] Derman E (1981). Isolation of a cDNA clone for mouse urinary proteins: age- and sex-related expression of mouse urinary protein genes is transcriptionally controlled. Proc Natl Acad Sci U S A..

[93] Shahan K, Denaro M (1987). Expression of six mouse major urinary protein genes in the mammary, parotid, sublingual, submaxillary, and lachrymal glands and in the liver. Mol Cell Biol..

[94] Stopka P (2016). On the saliva proteome of the Eastern European house mouse (Mus musculus musculus) focusing on sexual signalling and immunity. Sci Rep..

[95] Kuser PR (2001). The X-ray structure of a recombinant major urinary protein at 1.75 A resolution. A comparative study of X-ray and NMR-derived structures. Acta Crystallogr D Biol Crystallogr..

[96] 2018. Available from: http://useast.ensembl.org/Mus_musculus/Location/View?db=core;g=ENSMUSG00000078683;r=4:60498012-60501960 . Accessed 13 Oct 2018.

[97] Feyereisen R (2011). Arthropod CYPomes illustrate the tempo and mode in P450 evolution. Biochim Biophys Acta..

[98] Johnson RN (2018). Adaptation and conservation insights from the koala genome. Nat Genet..

[99] Jackson BC (2011). Update of the human secretoglobin (SCGB) gene superfamily and an example of ‘evolutionary bloom’ of androgen-binding protein genes within the mouse Scgb gene superfamily. Hum Genomics..

[100] Cavaggioni A, Mucignat-Caretta C (2000). Major urinary proteins, alpha(2U)-globulins and aphrodisin. Biochim Biophys Acta..

[101] Zhang ZD (2010). Identification and analysis of unitary pseudogenes: historic and contemporary gene losses in humans and other primates. Genome Biol..

[102] Young JM, Trask BJ (2007). V2R gene families degenerated in primates, dog and cow, but expanded in opossum. Trends Genet..

[103] Chamero P (2007). Identification of protein pheromones that promote aggressive behaviour. Nature..

[104] Nelson AC (2015). Protein pheromone expression levels predict and respond to the formation of social dominance networks. J Evol Biol..

[105] Hurst JL, Beynon RJ (2004). Scent wars: the chemobiology of competitive signalling in mice. Bioessays..

[106] Hurst JL (2001). Individual recognition in mice mediated by major urinary proteins. Nature..

[107] Deisig N (2014). Responses to pheromones in a complex odor world: sensory processing and behavior. Insects..

[108] Sengupta S, Smith DP, Mucignat-Caretta C (2014). How drosophila detect volatile pheromones: signaling, circuits, and behavior. Neurobiology of Chemical Communication.

[109] Hurst JL (1998). Proteins in urine scent marks of male house mice extend the longevity of olfactory signals. Anim Behav..

[110] Thonhauser KE (2013). Scent marking increases male reproductive success in wild house mice. Anim Behav..

[111] Janotova K, Stopka P (2011). The level of major urinary proteins is socially regulated in wild Mus musculus musculus. J Chem Ecol..

[112] Papes F, Logan DW, Stowers L (2010). The vomeronasal organ mediates interspecies defensive behaviors through detection of protein pheromone homologs. Cell..

[113] Roberts SA (2010). Darcin: a male pheromone that stimulates female memory and sexual attraction to an individual male's odour. BMC Biol..

[114] Roberts SA (2012). Pheromonal induction of spatial learning in mice. Science..

[115] Kimoto H, Haga S, Sato K, Touhara K. Sex-specific peptides from exocrine glands stimulate mouse vomeronasal sensory neurons. Nature. 2005;437(7060):898-901. 10.1038/nature04033 16208374

[116] More L (2006). Mouse major urinary proteins trigger ovulation via the vomeronasal organ. Chem Senses..

[117] Cheetham SA (2009). Limited variation in the major urinary proteins of laboratory mice. Physiol Behav..

[118] Sherborne AL (2007). The genetic basis of inbreeding avoidance in house mice. Curr Biol..

[119] Xiao Y (2013). Circulating lipocalin-2 and retinol-binding protein 4 are associated with intima-media thickness and subclinical atherosclerosis in patients with type-2 diabetes. PLoS One..

[120] De la Chesnaye E (2015). Lipocalin-2 plasmatic levels are reduced in patients with long-term type-2 diabetes mellitus. Int J Clin Exp Med..

[121] Xu A, Tso AW, Cheung BM, Wang Y, Wat NM, Fong CH, Yeung DC, Janus ED, Sham PC, Lam KS. Circulating adipocyte-fatty acid binding protein levels predict the development of the metabolic syndrome: a 5-year prospective study. Circulation. 2007;115(12):1537-1543. 10.1161/CIRCULATIONAHA.106.647503 17389279

[122] Baur JA, Pearson KJ, Price NL, Jamieson HA, Lerin C, Kalra A, Prabhu VV, Allard JS, Lopez-Lluch G, Lewis K, Pistell PJ, Poosala S, Becker KG, Boss O, Gwinn D, Wang M, Ramaswamy S, Fishbein KW, Spencer RG, Lakatta EG, Le Couteur D, Shaw RJ, Navas P, Puigserver P, Ingram DK, de Cabo R, Sinclair DA (2006). Resveratrol improves health and survival of mice on a high-calorie diet. Nature..

[123] Zhou Y, Rui L (2010). Major urinary protein regulation of chemical communication and nutrient metabolism. Vitam Horm..

[124] Dhahbi JM (2004). Temporal linkage between the phenotypic and genomic responses to caloric restriction. Proc Natl Acad Sci U S A..

[125] Miller RA (2002). Gene expression patterns in calorically restricted mice: partial overlap with long-lived mutant mice. Mol Endocrinol..

[126] Giller K (2013). Major urinary protein 5, a scent communication protein, is regulated by dietary restriction and subsequent re-feeding in mice. Proc Biol Sci..

[127] Zhou Y, Jiang L, Rui L (2009). Identification of MUP1 as a regulator for glucose and lipid metabolism in mice. J Biol Chem..

[128] De Giorgio MR, Yoshioka M, St-Amand J (2009). Feeding induced changes in the hypothalamic transcriptome. Clin Chim Acta..

[129] van Schothorst EM (2006). Adipose gene expression response of lean and obese mice to short-term dietary restriction. Obesity (Silver Spring)..

[130] Maxwell AI, Morrison GM, Dorin JR (2003). Rapid sequence divergence in mammalian beta-defensins by adaptive evolution. Mol Immunol..

[131] Kachroo AH (2015). Evolution. Systematic humanization of yeast genes reveals conserved functions and genetic modularity. Science..

